# Mpox 2022 to 2025 Update: A Comprehensive Review on Its Complications, Transmission, Diagnosis, and Treatment

**DOI:** 10.3390/v17060753

**Published:** 2025-05-25

**Authors:** Rajesh Yadav, Anis Ahmad Chaudhary, Ujjwal Srivastava, Saurabh Gupta, Sarvesh Rustagi, Hassan Ahmed Rudayni, Vivek Kumar Kashyap, Sanjay Kumar

**Affiliations:** 1 Sharda School of Allied Health Sciences, Sharda University, Greater Noida 201310, India; rajesh.yadav1@sharda.ac.in (R.Y.);; 2 Department of Biology, College of Science, Imam Mohammad Ibn Saud Islamic University (IMSIU), Riyadh 11623, Saudi Arabia; 3 Department of Biotechnology, GLA University, Mathura 281406, India; 4 Department of Food Technology, School of Agriculture, Maya Devi University, Dehradun 248011, India; 5 South Texas Center of Excellence in Cancer Research, School of Medicine, University of Texas Rio Grande Valley, McAllen, TX 78504, USA; 6 Division of Cancer Immunology and Microbiology, Medicine and Oncology Integrated Service Unit, School of Medicine, University of Texas Rio Grande Valley, McAllen, TX 78504, USA; 7 Biological and Bio-Computational Laboratory, Sharda School of Bio-Sciences and Technology, Sharda University, Greater Noida 201310, India; 8 DST-FIST Facility, Sharda University, Greater Noida 201310, India; 9 Centre of Excellence in Artificial Intelligence in Medicine, Imaging and Forensic, Sharda University, Greater Noida 201310, India

**Keywords:** monkeypox (Mpox), pathology, complication, virus replication, epidemiology, diagnosis and treatment of Mpox

## Abstract

Monkeypox virus (Mpox virus) is a zoonotic orthopoxvirus that has gained increased global attention due to recent outbreaks. The current review reports the latest update of Mpox cases from 25 February 2022 to 29 April 2025. It also evaluates the possible major complications in human life caused by Mpox. In early 2022, more than 40 countries reported Mpox outbreaks. As of 12 June 2024, the global case count for the 2022–2023 Mpox outbreak was 97,281 confirmed cases, in 118 countries. The World Health Organization (WHO) declared the Mpox virus, a zoonotic disease, a public health emergency of international concern (PHEIC) on 14 August 2024. Mpox symptoms include fever, headache, muscle pain, and face-to-body rashes. The review also highlights Mpox virus replication, genomics, pathology, transmission, diagnosis, and antiviral therapies. The 2022 outbreak is also discussed in detail. The coinfection of HIV in patients infected with Mpox is also discussed. The evolving Mpox epidemiology has raised concerns about the disease’s increasing spread in non-endemic countries, emphasizing the urgent need for control and prevention. The discussion on preventive measures, including vaccination, suggests that cross-protection against Mpox may be possible using orthopoxvirus-specific antibodies. Although there are no specific antiviral drugs available, certain drugs, such as tecovirimat, cidofovir, and ribavirin, are worth considering.

## 1. Introduction

**Figure 1:**
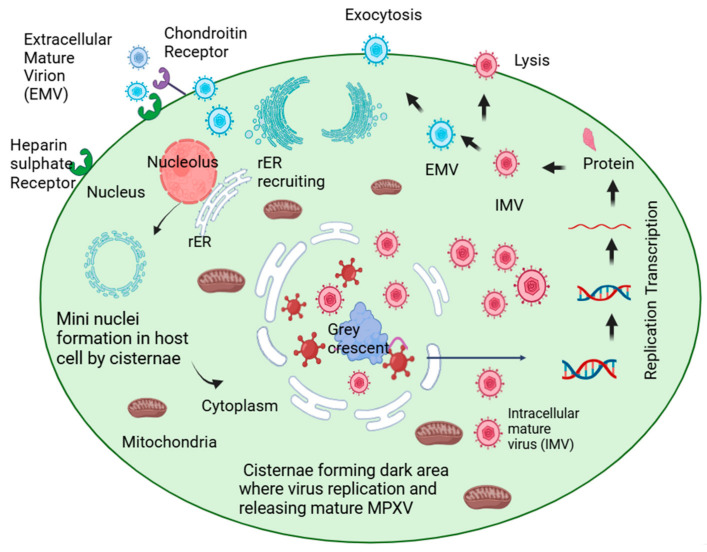
The schematic representation of intracellular mature virus releasing in a host cell, the cisternae of rough endoplasmic reticulum (rER) assemble and form mini nuclei in a host cell, and a large number of mitochondria are found near the mini nuclei. The replication of viral DNA occurs and forms m-RNA, which further forms viral protein, which is covered with an envelope and forms an intracellular mature virus.

Monkeypox is a viral disease caused by the Mpox virus. According to the latest report (29 April 2025) of the World Health Organization (WHO) globally, 137,892 confirmed cases of Mpox have been reported from 1 January 2022 to 31 March 2025. Out of these, 317 deaths were confirmed across 132 countries [1]. Mpox virus is an enveloped double-stranded DNA with around 200 kb of nucleotides, which is brick-shaped and has a tubule-like structure on its surface [2]. Additionally, it has a dumbbell-shaped core structure that measures up to 200 to 250 nm. This virus belongs to the genus Orthopoxvirus (OPXV) and the family of *poxviridae* [3]. Mpox resembles smallpox, and the disease is spread via zoonosis. There are two clades of Mpox: clade I (formerly the Central African clade or the Congo Basin clade) and Clade II (formerly the West African clade). Both have a sequence similarity of about 95%. The fatality rate of clade I is three times higher than that of clade II [4]. Mpox has a linear DNA genome that contains around 190 non-overlapping open reading frames (ORFs) [5]. Mpox genomes contain a highly conserved central region, while the terminal region is highly variable, having inverted terminal repeats (ITR) [6]. All Mpox viruses have four ORFs in the ITR region [5,7]. The gene loss at the terminal region acts as a driving force for the evolution of the new Mpox. The ultrastructural analysis study in 2022 revealed that virus replication occurs in the mini-nucleus, which is reorganized by endoplasmic reticulum cisternae in the host cell. The early mRNA and late mRNA form in the mini nucleus, which consecutively form the early protein and the late protein. As a result, an intracellular mature virus (IMV) is released in the cytoplasm and comes outside of the cell by exocytosis to form an enveloped extracellular virus or extracellular mature virus (EMV), which is ready to infect another host cell [8] (Figure 1). Mpox is not considered a sexually transmitted disease, although the Mpox virus DNA was detected in semen and seminal fluid samples in Italy and Germany in the 2022 global outbreak. Despite various studies on the Mpox virus, the anal or vaginal route can be considered a mucosal membrane route [9]. Virus mutation and the re-emergence of pathogens with unusual transmission routes may enhance infection and increase the possibility of an epidemic.

Since 2022, two major Mpox outbreaks have prompted the World Health Organization (WHO) to declare public health emergencies of international concern, first in July 2022 and again in August 2024. The initial global spread, attributed to a clade 2b strain, resulted in roughly 100,000 cases and 200 fatalities [10]. Subsequently, a new clade, 1b, emerged, leading to a second surge. As of December 2024, this recent outbreak has seen over 55,000 reported or suspected cases and approximately 1000 deaths, primarily in the Democratic Republic of Congo and neighboring nations such as Burundi, Rwanda, Uganda, and Angola [11]. Isolated cases of clade 1b have also been documented in countries including the UK, Sweden, Germany, Belgium, France, the USA, Canada, and Thailand. The details of the outbreaks of 2024 and 2022 are as follows.

### 1.1. The 2024 Outbreak

The Mpox clade 1b variant, initially detected in the Sud Kivu area of the Democratic Republic of Congo (DRC), has emerged as a major public health threat due to its increased ability to spread between people [12]. Genetic investigation of clade 1b has uncovered numerous mutations of Mpox. The genetic makeup of clade 1b shows several mutations in genes related to the enzyme apolipoprotein B mRNA editing catalytic polypeptide-like 3 (APOBEC3) cytosine deaminase. This enzyme’s involvement is often indicative of sustained human-to-human transmission [13]. These genetic alterations distinguish clade 1b from the traditionally circulating clade I variant, enabling more efficient spread through close physical contact, including sexual contact. 

Genetic changes, particularly those linked to APOBEC3, are thought to contribute to clade 1b’s enhanced transmissibility.

Since its initial identification, this variant has expanded its reach within the DRC to other provinces and has also been reported in neighboring nations like Rwanda, Uganda, and Kenya, signifying a considerable geographical expansion of the outbreak [14,15].

The Democratic Republic of Congo was the epicenter and reported 3235 Mpox infections and 19 deaths as of 11 August 2024 [16]. The emergence of the clade 1b Mpox in neighboring Central and East African countries has raised significant concerns about the potential for a wider regional epidemic. Burundi had recorded 545 Mpox alerts by 17 August 2024, and 142 were confirmed positive for the clade Ib Mpox variant. Rwanda confirmed its initial two Mpox infections on 24 July 2024, prompting the Ministry of Health to declare an outbreak on July 27. By 7 August 2024, Rwanda had reported a total of four confirmed cases of the clade 1b variant of Mpox, with no associated deaths. In Uganda, initially, six suspected cases were identified in June 2024, and out of those six, two were confirmed later. As of 13 August 2024, Kenya has identified 14 suspected cases, with one confirmed positive for clade Ib Mpox. To date, no deaths have been reported.

In August 2024, the clade 1b strain’s spread beyond Africa was confirmed for the first time [15]. The virus has also extended its reach to countries in East Africa, Europe, and Asia, underscoring its capacity for worldwide spread [16]. Sweden reported a case involving a traveler returning from an affected region, demonstrating the pivotal role of international travel in spreading infectious diseases globally [17]. This event prompted urgent calls for governments to enhance border checks, implement targeted surveillance, and foster international collaboration to control cross-border disease transmission. On 22 August 2024, Thailand reported its initial Mpox case of a European man with a recent travel history to Africa [18]. Likewise, India’s identification of its first imported clade 1b Mpox case in Kerala highlights the potential for international travel to contribute to the dissemination of this variant.

From November 2024 to 12 February 2025, four cases of clade Ib were detected. In the United States, the first confirmed case of clade Ib Mpox occurred in California in November 2024, following travel to an affected region. A second case was identified in Georgia on 14 January 2025, involving a traveler from a country with ongoing Mpox transmission. Subsequent cases were reported in New Hampshire on 7 February 2025 and in New York on 12 February 2025. These four cases represent independent events, with no epidemiological links established and no evidence of further transmission [19]. The 2024 outbreak emphasized the importance of aligning travel health policies with robust outbreak management strategies to minimize risks from rapidly spreading pathogens [15].

A significant public health crisis unfolded between 2022 and 31 December 2024, with global records documenting over 118,777 Mpox infections [20]. Between 2022 and 2024, the different regions of the Americas documented 67,220 confirmed Mpox cases, resulting in 151 deaths across 31 countries and territories [20]. The majority of these cases occurred in 2022 (57,616, representing 85.7% of the total), followed by a substantial decline in 2023 (4056 cases, 6%). A minor increase was observed in 2024, with 5548 cases (8.3%) [20]. The World Health Organization (WHO) has determined that the highly contagious clade 1b strain is the primary driver of this outbreak, characterized by severe symptoms and increased mortality [21]. The most affected areas that reported a large number of infections were East, Central, West, and Southern Africa, specifically, Burundi, Kenya, Rwanda, Uganda, the Central African Republic (CAR), Ivory Coast, and South Africa [22]. The swift dissemination of the virus in these areas underscores the critical need for collaborative containment efforts and enhanced healthcare systems to effectively address such epidemics.

### 1.2. The 2022 Outbreak

Before 2022, Mpox cases were documented within the African continent. The first case of the 2022 outbreak of the Mpox outside the African continent was recorded on 6 May 2022 in the United Kingdom [23]. A total of 366 Mpox cases were confirmed in the United Kingdom (UK) until 10 June 2022 [24,25]. On 18 May 2022, Portugal reported 14 Mpox infections, Spain reported seven, and Canada reported 13. The first Mpox cases in Belgium, Sweden, and Italy were confirmed on 19 May 2022. France, Germany, Australia, and the Netherlands each reported their initial Mpox cases on 20 May 2022. Switzerland and Israel each confirmed their initial Mpox cases on May 21. By 24 May 2022, 19 countries had reported cases of Mpox. Among these, the United Arab Emirates identified its first case in late May, involving a 29-year-old female traveler from West Africa. Slovenia also confirmed its initial infection. Denmark’s first case was linked to an individual returning from the Canary Islands. In Canada, Quebec reported fifteen confirmed cases on 24 May, the same day the Czech Republic confirmed its first case [24]. The study published in the New England Journal on 21 July 2022 reported a total of 528 Mpox infections between 27 April and 24 June 2022, at 43 sites within 16 countries [26].

By 27 May 2022, Portugal had confirmed 96 Mpox cases, and the information regarding demographics, clinical presentation, and exposure was collected from all 96 cases via face-to-face and telephone interviews using standardized case investigation forms. A subset of 27 confirmed cases underwent more detailed investigation, and their sociodemographic and clinical characteristics, along with available laboratory and epidemiological findings, are summarized in a previous study published in Eurosurveillance in June 2022 [27]. The majority of cases (25 cases) of the 2022 Portugal outbreak resided in the Lisbon and Tagus Valley (LVT) region, with single cases in the North and Algarve regions. All cases were male, ages ranging from 22 to 51 years (majority 30–39). Considering exposures within the 21 days preceding symptom onset, few (1/10) reported contact with individuals exhibiting similar symptoms, and some had a history of international travel (4/27).

Most cases were identified as men who have sex with men (MSM) (18/19), while one reported sex with only women. During the 21 days before symptom onset, most cases with available data (14/16) reported multiple sexual partners. Six cases reported attending a sauna in the LVT region, one frequented a UK sauna, and four reported international travel—three reported contacts with animals (two with cats and one with pigs). Common symptoms included exanthema (fourteen), inguinal lymphadenopathy (fourteen), fever (thirteen), and genital ulcers (six). Fourteen cases were HIV-positive. Three cases required hospitalization, two of whom were later discharged. No deaths were reported by 27 May 2022. One middle-aged case reported prior smallpox vaccination [27].

In May 2022, the World Health Organization (WHO) declared a global health emergency for a previous Mpox outbreak caused by clade IIb of the virus. Almost 48 cases of Mpox were reported from different regions of Latin America, such as Brazil, Argentina, Peru, Venezuela, Mexico, Colombia, and Chile, by 28 June 2022 [28]. In July 2022, the multi-country Mpox outbreak was declared a Public Health Emergency of International Concern (PHEIC) due to its rapid spread through sexual contact in regions previously unaffected by the virus. This declaration was lifted in May 2023 following a sustained global decline in cases. The outbreak had infected 87,000 people and resulted in 140 deaths. The WHO attributed the successful containment to a combination of vaccination and public health information campaigns [29]. Most of these countries have set up their epidemiological centers for the identification of these viruses. According to the Centers for Disease Control and Prevention, as of 7 February 2024, the maximum number of reported cases in the USA is 31,894, followed by Brazil (10,967), Spain (7752), and France (4171).

**Figure 2:**
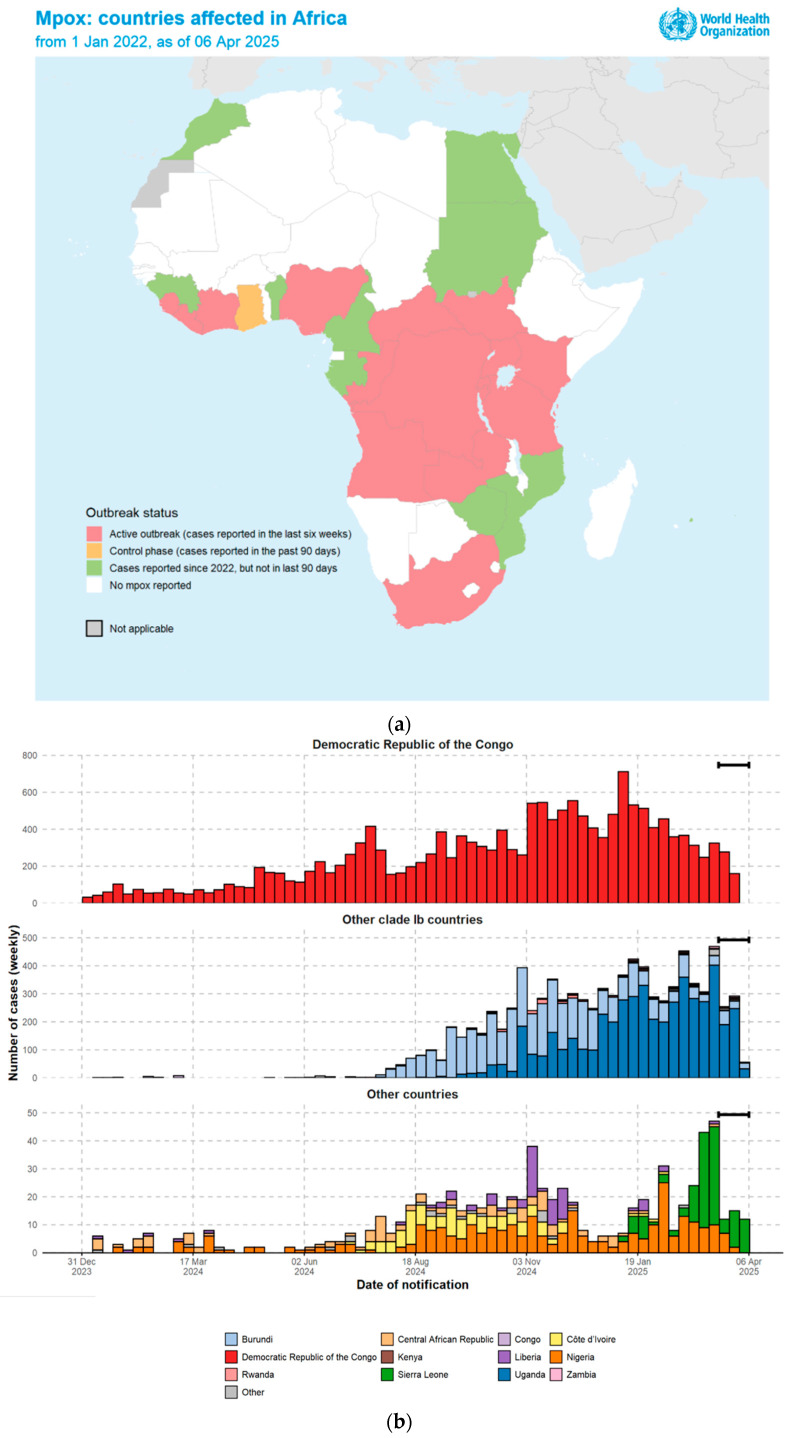
(**a**) Shows the geographical distribution of Mpox in countries affected in Africa. Figure credit source: WHO. (**b**) Trends in confirmed Mpox cases in Africa (data as of 6 April 2025). Updated data from 1 January 2024 to 6 April 2025. The data shown here include laboratory-confirmed cases only. In the Democratic Republic of the Congo, a small number of cases are not represented on the epidemic curves due to missing data. Brackets at the end of the curve indicate potential reporting delays in recent weeks of data. Figure and data source: WHO.

The geographical distribution of the Mpox in countries affected in Africa is illustrated in Figure 2a, while the recent trends of Mpox cases in Africa are shown in Figure 2b.

## 2. Host Reservoirs and Transmission

Mpox is a zoonotic disease; Central African rodents and primates are the natural hosts of Mpox. The Mpox virus is primarily transmitted from animals to humans through close contact, such as bites, scratches, or exposure to the animal’s rash, as well as through exposure to their bodily fluids or consumption of improperly cooked meat [30]. Transmission can also occur via contaminated materials (fomites) like bedding or clothing. Historically, human-to-human transmission has been less frequent.

A 2018 case study reported that a man traveling from Nigeria to the UK on September 6th presented with a maculopapular rash, fever, and lymphadenopathy and was subsequently diagnosed with Mpox. One of the three attending healthcare workers developed symptoms, was isolated, and had 134 contacts traced and monitored. Four of these contacts later became ill. This incident demonstrates human-to-human transmission of Mpox from a traveler returning from Nigeria to healthcare staff [31].

Historically, Mpox infection was primarily linked to animal contact or travel to endemic regions. However, the transmission pattern shifted in the 2022 outbreak, with most cases now attributed to sexual contact. Over the last two years (2020–22), predominantly gay and bisexual men have been affected [32]. The study reveals that the risk of public transmission was very low because Mpox virus transmission from human to human requires close contact. Human-to-human transmission of the Mpox virus occurred through direct contact through blood transfusion, respiratory droplet infection, sharing of foods, clothes, and bedding, and sexual activity, especially with male-to-male sex [33].

**Figure 3:**
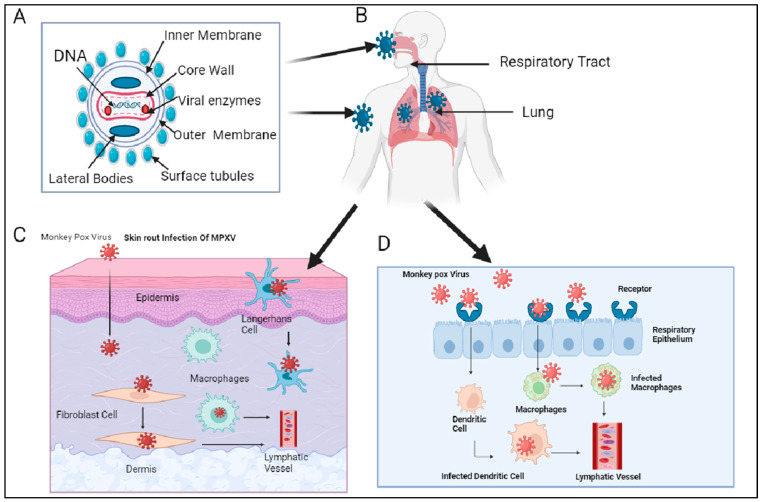
A schematic diagram of the Mpox virus and its entry route in humans was created by biorender.com. (**A**) Shows the structure of the Mpox virus. (**B**) Shows virus entry in humans through different routes. (**C**) Shows the entry of the Mpox virus through the skin route. (**D**) Shows the entry of the Mpox virus through respiratory epithelium in humans.

The Mpox virus may be transmitted from the mother to the fetus during pregnancy, and that type of transmission is called vertical transmission [34]. The different proposed model admits that during the entry of the Mpox virus through the respiratory epithelium in humans, the Mpox virus enters dendritic cells and macrophages, which then enter lymphatic vessels and the lymphatic system. In the same way, when the Mpox virus infects the skin epidermis, it first infects fibroblast cells and Langerhans cells, which move in lymphatic vessels and then into the lymphatic system. In this way, it spreads all over the body and infects the liver, which may cause serious liver problems (Figure 3).

## 3. Complications Associated with Mpox

The various complications that have been observed in Mpox-infected persons are the following.

### 3.1. Neurological and Psychiatric Complications

Mpox infections are associated with potential neurological and mental health complications. Research conducted in Nigeria in 2020 indicated that a quarter of hospitalized Mpox patients experienced psychiatric symptoms, such as anxiety, depression, and suicidal ideation [35]. However, it is difficult to isolate these symptoms from the psychological impact of hospitalization and isolation. Neurological issues, including seizures, confusion, and encephalitis, were observed in Mpox patients during the 2022 outbreak, with reported occurrences of 2.7%, 2.4%, and 2%, respectively [36]. Furthermore, cerebrospinal fluid (CSF) analysis revealed the presence of Mpox-specific IgM antibodies. Studies indicate that neuropsychiatric symptoms are common in 50% of individuals with Mpox infection. Badenoch and colleagues suggest that symptoms occur in more than half of cases [36]. These symptoms may arise from the infection itself or the experience of quarantine [37]. Additionally, research conducted in Iraq has revealed widespread anxiety among the general population concerning the ongoing Mpox outbreak [38]. This research sought to evaluate public awareness, perceptions, and anxiety related to the multi-country Mpox outbreak within the Kurdistan region of Iraq. A digital survey utilizing a convenience sampling approach was conducted from 27 to 30 July 2022. The study included 510 participants. Overall, the population demonstrated a moderate understanding of Mpox, held a neutral attitude toward it, and experienced a moderate level of anxiety. Statistical analysis revealed that demographic factors, including gender, religion, education level, and place of residence, significantly impacted awareness and anxiety levels. Gender and the residential area also played a significant role in shaping attitudes toward Mpox [38].

### 3.2. Dermatological Complications

A common characteristic of Mpox infection is the appearance of skin lesions, particularly in the anogenital region. Skin lesions present as a rash that progresses through distinct stages: initially flat (macular), then raised (popular), and subsequently fluid-filled (vesicular). These lesions typically resolve with crusting within three weeks [39]. A characteristic Mpox rash begins with small, flat (macules) or raised (papules) lesions, typically 0.5 to 1 cm in diameter. These lesions progress over two to three weeks, evolving into fluid-filled vesicles and pus-filled pustules, often with a central depression (umbilication), and eventually form crusts. The vesicles and pustules themselves are generally spherical, ranging from 0.5 to 2 cm in diameter, and are notable for their firm texture, deep skin involvement, and well-defined edges [40].

A hallmark of Mpox is a unique rash characterized by painful or itchy maculopapular lesions that progress into a vesiculopustular eruption [41]. Typically, the rash emerges within one to three days following the onset of initial symptoms. However, variations exist, with some individuals experiencing the rash more than three days after fever onset or concurrently with the fever. Lesions commonly manifest on the face, trunk, extremities, genital region, scalp, palms, and soles. The rash often begins on the face and then spreads outward (centrifugally) to the limbs, palms, soles, and mucous membranes of the mouth, eyes, and genitals. A higher concentration of lesions is generally observed on the face and limbs [42]. In some cases, oral lesions, known as enanthems, may precede the appearance of the skin rash. A comprehensive review of available research indicates that sore throat is the most frequently reported oral symptom of Mpox, while ulceration is the most common oral or peri-oral sign [43].

In one study of 54 patients, all presented with skin lesions, with a significant majority (94%) exhibiting anogenital involvement [44]. A large portion of these patients (89%) reported lesions at multiple anatomical sites, and a smaller percentage (7%) had lesions in the oropharyngeal area. Additionally, a substantial number of patients experienced fatigue (67%) and fever (57%), while a minority (18%) showed no initial symptoms. In some cases, genital ulcers were observed to be painless, accompanied by bilateral inguinal lymphadenopathy, resembling the presentation of primary syphilis [45].

A separate international study, tracking 528 Mpox infections in 2022, also identified anogenital lesions as a prevalent symptom. This study found that 73% of patients had anogenital lesions, and 41% presented with mucosal lesions. A rash was observed in 95% of patients, with other frequently reported symptoms including fever (62%), lymphadenopathy (56%), lethargy (41%), muscle aches (31%), and headaches (27% [26]). Due to the varied and evolving clinical presentation of Mpox, especially as seen in the 2022 outbreak, medical professionals, particularly dermatologists, are advised to exercise a high degree of vigilance when evaluating patients with suspected Mpox infection.

The other study, conducted in 101 patients from thirteen countries, shows that 54% of cases have been found with skin lesions. During the initial five days of infection, papules (36%), vesicles (17%), and pustules (20%) were the most frequently observed skin lesions. From days 6–10, pustules became the dominant lesion (36%), followed by erosions/ulcers (27%) and crusts/scabs (24%). After day 11, crusts/scabs were the primary skin manifestation [46].

While monkeypox treatment remains largely symptomatic due to the lack of specific antiviral medications, a novel therapeutic approach combining antimicrobial photodynamic therapy (aPDT) and photobiomodulation therapy (PBMT) successfully treated a large facial cutaneous lesion, highlighting its potential as a promising intervention [47].

### 3.3. Complications Related to the Coinfection of Mpox Virus and HIV

The COVID-19 pandemic, which began in 2020, demonstrated the rapid emergence of viral diseases and the potential for global spread, highlighting the ongoing threat of Mpox. Mpox may be more lethal for immunocompromised people, such as those infected with the human immunodeficiency virus (HIV). The coinfection of Mpox virus and HIV was first identified and diagnosed in Latin America [48]. In 2022, the recent coinfection of Mpox and acute HIV was also confirmed in a 24-year-old male with outstretched papules throughout the trunk, face, and genital area [49]. A 2023 review in *Vaccine* reported 6345 confirmed Mpox cases across 53 studies, with a 40.32% HIV co-infection rate [50]. According to WHO, 51% (13,769/26,992) of confirmed cases of Mpox have HIV [51]. The outbreak of Mpox in multiple countries was reported during 2022–24. According to the WHO report, the maximum number of confirmed cases came from the European region (86%), followed by America (11%), the African region (2%), and the Eastern Mediterranean region (1%) [52]. Co-infection with HIV increases the chance of infection in an uninfected person by enhancing the concentration of HIV on genital organs (lesions) and genital secretions or by increasing both factors [53,54]. Individuals living with HIV, especially those with compromised immune systems characterized by low CD4 cell counts (<500 cells per mm^3^), demonstrate an increased vulnerability to severe Mpox complications and mortality compared to those without HIV. The immunodeficiency resulting from advanced HIV infection elevates the susceptibility to severe Mpox manifestations, which can include necrotizing skin lesions, pulmonary involvement, secondary infections, and sepsis [55].

This study analysed 382 Mpox cases, comprising 367 cisgender males, four cisgender females, and ten transgender females. The median age of the participants was 35 years, with an interquartile range (IQR) of 30 to 43 years. At the time of Mpox diagnosis, 349 individuals (91%) were known to be living with HIV. Among those with HIV, 228 (65%) were adhering to antiretroviral therapy (ART). Concurrent opportunistic infections were observed in 32 participants (8%). The median CD4 cell count was 211 cells/mm^3^ (IQR 117–291). Specifically, 85 individuals (22%) had CD4 counts below 100 cells/mm^3^, and 94 individuals (25%) had counts between 100 and 200 cells/mm^3^. An undetectable viral load was present in 193 participants (51%) [55].

Severe Mpox complications were significantly more prevalent in individuals with CD4 counts below 100 cells/mm^3^ compared to those with counts exceeding 300 cells/mm^3^. These complications included the following: necrotizing skin lesions (54% vs. 7%), pulmonary involvement, sometimes with nodules (29% vs. 0%), and secondary infections and sepsis (44% vs. 9%).

Hospitalization was required for 107 participants (28%), with 27 (25%) of those hospitalized resulting in death. All fatalities occurred in individuals with CD4 counts below 200 cells/mm^3^. Within this group, mortality was higher among those with elevated HIV viral loads. Immune reconstitution inflammatory syndrome (IRIS) related to Mpox was suspected in 21 of the 85 individuals (25%) who initiated or restarted ART, with 12 (57%) of these cases resulting in death. Tecovirimat was administered to 62 participants (16%), while cidofovir or brincidofovir was used in seven participants (2%). Tecovirimat resistance was confirmed in three cases.

### 3.4. Complications Associated with Heart (Myocarditis)

Myocarditis, pericarditis, and myopericarditis have been rarely documented in Mpox infection. Two immunocompetent, unvaccinated adults in the U.S. developed monkeypox-related myocarditis. They were hospitalized and presented with cardiac symptoms (Chest pain) and elevated biomarkers. Oral tecovirimat and doxycycline were provided for the treatment of monkeypox, and they were discharged home after recovery without immediate complications. They received no specific treatment for myocarditis, given the rapid resolution of symptoms and normalization of troponin levels [56].

In a 2022 study, Thornhill et al. documented two instances of self-limiting myocarditis associated with monkeypox, where patients recovered within seven days without significant complications. One of these patients had a history of HIV with a normal CD4 count [26].

A wide range of viruses are known to cause myocarditis, such as Coxsackieviruses and adenoviruses [57]. Viral myocarditis can cause dilated cardiomyopathy. The most frequently observed pathological process involves lymphocytic myocarditis with myocyte necrosis, typically developing 10 to 14 days after the viral infection. The occurrence of myocardial involvement in orthopoxvirus infections was initially documented following smallpox vaccination in young military recruits, using live vaccinia-based vaccines [58]. The precise pathophysiological pathways leading to orthopox-induced myocarditis remain to be determined. Heart failure, arrhythmias, and other cardiovascular-related problems may also be possible in Mpox infection [59].

### 3.5. Hypotension Sepsis Complication

In severe cases, significant fluid loss due to fever, rash, and capillary permeability can occur. The associated systemic inflammatory response may also contribute to hypotension. These factors combine to reduce renal perfusion, potentially leading to acute kidney injury. Vigilant monitoring of urine output and blood chemistry is essential for early detection and to guide the necessity of intravenous fluid replacement.

While infrequent, secondary bacterial infections leading to sepsis have been documented in Mpox-infected patients with *Streptococcus pyogenes* [60]. Prompt identification of sepsis, utilizing standard diagnostic criteria, is essential. Treatment should adhere to established sepsis management protocols, including initiating broad-spectrum empirical antibiotics within one hour of diagnosis, rapid intravenous fluid resuscitation with ongoing response evaluation, vasoactive medication administration, and appropriate respiratory support [61].

### 3.6. Ocular Complication

Ocular symptoms are uncommon but can cause corneal scarring and vision loss [3]. Manifestations, potentially from self-inoculation, include conjunctivitis, blepharitis, and other ocular inflammations. The 2022 Mpox outbreak showed a significantly lower ocular involvement rate of approximately 1%, compared to 9–23% in prior outbreaks [62]. Mpox can present with a variety of ocular symptoms, including redness, frontal headaches, rashes around the eyes, tearing, discharge, and subconjunctival nodules. Less commonly, it may lead to more severe complications like keratitis, corneal ulcers, opacification, perforation, and vision loss. In the 2022 outbreak, these ocular symptoms have been observed less often and potentially with reduced severity. Though potential underreporting exists, observational studies suggest an ocular involvement rate of approximately 1%, significantly lower than the 9–23% incidence reported in previous outbreaks in endemic regions [62,63]. Newborns have been reported to experience ocular involvement with Mpox [64]. Additionally, there are documented cases (53-year-old patient in San Francisco, CA, USA) where ocular symptoms presented independently, without concurrent skin lesions [65].

Patients presenting with ocular Mpox symptoms should be referred for ophthalmologic evaluation. A slit-lamp examination and dilated fundoscopy are valuable tools for assessing the involvement of both anterior and posterior eye structures [66].

Retinol supplementation is advised. Supportive measures such as eye lubrication and saline compresses may provide relief. In cases of co-infection, ophthalmic antibiotics or antivirals may be necessary [67]. Patients should be instructed to avoid contact lens use [68]. Topical trifluridine, under ophthalmological guidance, can be considered for conjunctivitis and is recommended for keratitis management.

### 3.7. Fulminant Mpox

A severe, widespread, and destructive form of Mpox has been observed in individuals with advanced immunosuppression, such as those with CD4 counts below 100 cells/µL, high HIV viral loads, or hepatitis B co-infection. These cases show a resemblance to an AIDS-defining illness. Patients showed extensive, large necrotic skin and mucosal lesions that often merged [69]. Severe secondary bacterial infections were frequent. Complications included lung nodules, necrotic pulmonary masses, nodular hepatic lesions, respiratory insufficiency, and sepsis [70]. Immune reconstitution inflammatory syndrome (IRIS) was suspected in a quarter of patients initiating or resuming antiretroviral therapy post-diagnosis [71]. Mortality was reported in 15% of patients, primarily those with CD4 counts below 200 cells/µL [70].

In moderately to severely immunocompromised patients with uncontrolled viral dissemination, optimal immune function should be prioritized. Tecovirimat should be initiated promptly, and consider adding cidofovir or brincidofovir and vaccinia immune globulin. Prolonged treatment may be necessary. Wound care is essential, and fluid management may be required. Specialist consultation should be considered as needed. Close monitoring for complications is vital [72].

### 3.8. Complications Associated with Immune System

**Figure 4:**
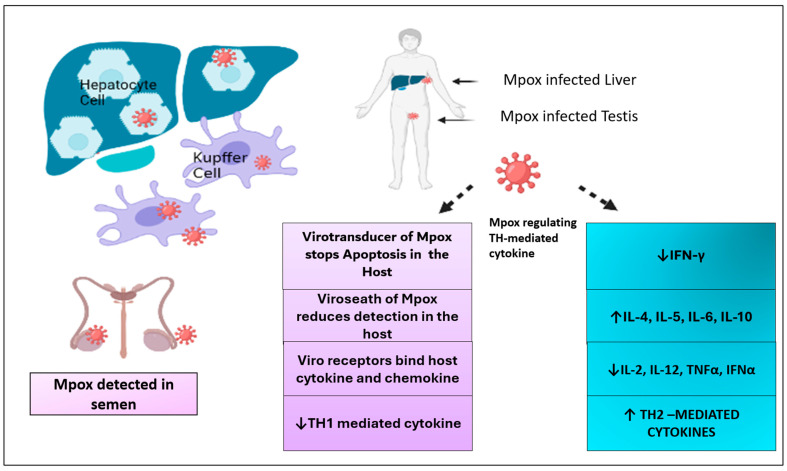
The schematic diagram shows an Mpox-infected liver and testis and its evading mechanism in host cells with cytokine storm production, representing increased Th2-mediated immune response and decreased TH1-mediated immune response.

Like COVID-19 infection, Mpox also creates cytokines inside the body [73]. Cytokines are Th2-mediated, and a large number of interleukins (IL-4, IL5, IL6, and IL10) are released, while TH1-mediated cytokines are released in a lower amount. Therefore, IFγ (interferon gamma), IL2, IL12, TNF-α, and IFN-α levels decreased after Mpox infection [74]. The human Mpox virus causes post-translational modification on the host cell. It downregulates those regulatory factors that increase histone expression while upregulating core histone proteins. The nucleosome formation of viruses depends on host nucleosome modification [75]. Mpox has a specific mechanism through which it defends itself via the immune reaction of the host. The intracellular protein of Mpox called Viro transducer stops apoptosis in the host cell and stops the host cell from responding against the virus [33,76]. In this way, these proteins evade the host’s immune system to allow for viral replication in the host, as shown in Figure 4. The Mpox virus uses various strategies in the host cell to escape from the host immune defense system. Innate immunity is the first line of defense and is operated by interferons (IFNs) [77]. Interferons prevent viral replication in host cells using direct or indirect pathways. There are three types of interferons: type 1, type 2, and type 3. IFNα, IFNβ, and IFNω are considered type I interferons. Type I interferons create antiviral activity in cells by activating Janus protein tyrosine kinase and signal transducers and activators of the transcription (JAK-STAT) pathway [78]. Mpox virus evades the immune response of the host in the following way: (a) An in vitro study in 2010 showed that Mpox viral protein B16 inhibits JAK-STAT induced by type I interferon [79]. (b) Another experiment performed in mice in 2004 found that homologs of D7L block interleukin (IL-18) [80]. The complement control protein (CCP) of Mpox inhibits the complement activation pathway, which is a key factor for viral defense [81]. Apart from it, many proteins of Mpox are responsible for evasion, including ankyrin-like protein, viral growth factor, IL-18 binding protein, apoptosis inhibitors, SPI-1, inhibitors of MHC class I molecules, inhibitors of IRF3 NF-kB activation, Inhibitor of IRF3 and IRF7 activation, inhibitors of MHC class II, CC, and CXC chemokine binding proteins.

## 4. Diagnosis of Mpox

PCR (polymerase chain reaction) is a gold standard molecular biology technique that detects the presence of Mpox viral DNA in samples such as blood, skin lesions, or respiratory secretions. PCR is a highly sensitive and specific method used for confirmatory diagnosis. Real-time PCR is the most common diagnostic procedure for the detection of Mpox. In the 2022–23 outbreak of Mpox, four novel molecular kits were used: The Novaplex MPXV Assay, the STANDARD M10 MPX/OPX, the Real Cycler MONK-UX/MONK-GX v.2, and the RealStar Orthopoxvirus PCR Kit 1.0, with an in-house PCR assay. A comparison study of diagnostic kits shows that sensitivity and specificity were 100% for the Novaplex MPXV Assay and RealStar Orthopoxvirus PCR Kit 1.0. The RealCycler MONK-UX/MONK-GX v.2 and STANDARD M10 MPX/OPX exhibited a slight decrease in the accuracy (97.3%) and diagnostic sensitivity (96.3%) [82].

The lab diagnosis of viral infections plays a crucial role in the detection of diseases and their treatment. Cell culture or tissue culture is considered to be the standard method for the isolation and cultivation of viruses, although this method is considered to be time-consuming and has a high risk of contamination [83]. The other standard methods for the detection of viruses are molecular and non-molecular techniques (immunofluorescence technique and ELISA), electron microscopy-based detection (TEM), radiation-based virus detection (X-ray diffraction technique and CT), nucleic acid amplification methods (NASBA, LAMP, RT-PCR), antigen-antibody complex-mediated detection of viruses (HAI), and CRISPR-CAS system-based detection [84]. Urine, saliva, semen, and rectal or genital samples can also be taken for investigative purposes, depending on the clinic [85].

CRISPR-based detection is also a rising diagnostic method in the field of virology. According to research conducted [86], a system was designed to detect the Mpox viral DNA using a fluorescence readout. In this study, it was shown that the G-quadruplex oligonucleotide of the target DNA, which was labeled with fluorescein (6-FAM) on the 3-prime end and black hole 1-quencher (BHQ-1) at the 5-prime end, produced a fluorescence signal when it was degraded via the CAS-12a-mediated collateral cleavage. This effect was not shown in the absence of the target DNA because CAS-12a is unable to cleave the labeled G-quadruplex oligonucleotide, and fluorescence quenching happens.

Pan-orthopoxvirus PCR/ESI-MS is another emerging method in the field of diagnostics; it can perform identification of all members of orthomyxovirus without sequencing by using the T5000 platform [87]. Here, A PCR method coupled with electrospray ionization mass spectrometry (PCR/ESI-MS) was employed to diagnose Mpox in spiked human samples and aerosol-infected cynomolgus macaque samples.

Loop-mediated isothermal amplification (LAMP) is another molecular diagnostic technique used in virology, offering a potential alternative to RT-PCR. This technique uses the displacement of the auto-cycling step of DNA synthesis by Bst DNA polymerase, which replicates the gene without the use of a light cycle [88].

## 5. Treatment of Mpox

The treatment of Mpox primarily focuses on managing symptoms and preventing complications. The symptoms, like fever, pain, and itching, can be treated with acetaminophen or ibuprofen. Antiviral medication such as tecovirimat has been used for some patients with Mpox, particularly those with severe illness or who are at high risk of complications. Other antiviral medications, such as brincidofovir and cidofovir, may be considered in certain situations, but their use is generally limited. Vaccines are also recommended for people at high risk of exposure. To prevent infection with the Mpox virus, avoid close skin contact and contact with contaminated materials (e.g., clothing, bedding). Most people with Mpox experience mild symptoms and recover without specific treatment. Severe cases and complications are more common in individuals with weakened immune systems, children, and pregnant women.

### 5.1. Vaccine Strategies Against Mpox

Almost a thousand cases have been reported from different parts of the world since April 2022, mostly from non-endemic countries; therefore, it has become necessary to understand the possible risk factors associated with this virus. Viral inactivation is a crucial step to prevent the spreading of Mpox; among the methods of viral inactivation, heat inactivation is one of the methods of viral inactivation.

According to Batéjat Christophe et al., the Mpox virus was inoculated on two growth media named FCS and VTM and then subjected to heat inactivation at different temperatures over a period. This study showed that Mpox got inactivated in less than 5 min if heated at 70 °C and less than 15 min if heated at 60 °C, and there was no difference between clade I and clade II [89].

The United States of America currently has three vaccines against smallpox: JYNNEOS^TM^, also known as IMVAMUNE, ACAM2000^®^, and Aventis Pasteur smallpox vaccine (APSV) [90]. This vaccine JYNNEOS^TM^ has been prepared from the modified Ortho poxvirus vaccinia Ankara-Bavarian Nordic strain and is attenuated in nature and non-replicating.

In August 2007, the FDA approved ACAM2000^®^ for the prevention of smallpox. The ACAM2000^®^ vaccine is also a live vaccine but shows some differences with JYNNEOS™. ACAM2000^®^ is a replication-competent vaccinia virus, whereas JYNNEOS™ is a replication-deficient modified vaccinia Ankara virus. ACAM2000^®^ shows some major cutaneous reactions, but JYNNEOS™ does not show cutaneous reactions. Under Emergency Use Authorization (EUA), the Aventis Pasteur Smallpox Vaccine (APSV) can be used, but its effectiveness against Mpox is still unknown [90].

The FDA (Food and Drug Administration) approved the use of the JYNNEOS™ vaccine in 2019 to prevent smallpox and Mpox [91]. Originally, the FDA developed these vaccines for the subcutaneous route in a two-dose series (0.5 mL per dose, administered 4 weeks apart). However, due to overwhelming demand, the FDA issued an Emergency Use Authorization (EUA) on 9 August 2022 for the intradermal route in a two-dose series (0.1 mL per dose, administered four weeks apart).

It was also found that full vaccination with the vaccine through subcutaneous, intradermal, and heterologous routes had an adjusted VE of 88.9% (95% CL, 56.0 to 97.2), 80.3% (95% CL, 22.9 to 95.0), and 86.9% (95% CL, 69.1 to 94.5), respectively. These vaccines are administered to persons who are either suffering from Mpox or have been previously exposed to such patients of Mpox. These vaccines (JYNNEOS™) are also offered to persons who are at high risk of getting infected by Mpox, such as gay, bisexual, MSM, transgender, non-binary, or gender non-conforming persons and HIV patients [92].

Huber et al. have shown the effectiveness of the vaccine on patients suffering from Mpox and others with MSM (men who have sex with another man), aged 18–49. A total of 309 infected patients (Mpox or MSM) were included in this study and found that the adjusted vaccine efficiency (VE) was 75.2% (95% confidence interval (CL) 61.2 to 84.2) in patients with a single dose (partial vaccination) of vaccine and 85.9% (95% CL, 73.8 to 92.4) in patients with full (two doses) vaccination [93].

Lauren Pischel quantified the vaccine efficacy of third-generation Mpox vaccines (MVA-BN, LC16m8, OrthopoxVac) in preventing infection, hospitalization, and death among the global population [94]. They compared vaccinated individuals to those unvaccinated or vaccinated with other vaccines. Additionally, they analyzed vaccine efficacy based on the number of vaccine doses (one or two) and whether the vaccine was administered as post-exposure prophylaxis (PEP). A comprehensive literature search yielded 11,892 initial records, with an additional 3223 identified through citation tracking. Thirty-three studies focused on third-generation Mpox vaccines, predominantly MVA-BN (32 studies). Two additional studies reanalysed existing data. The majority of these studies cantered on gay, bisexual, or other men who have sex with men aged 18–49, conducted between May and October 2022.

Vaccine efficacy for a single dose of MVA-BN was estimated at 76% (95% CI: 64–88%) across twelve studies. For two doses, the vaccine efficacy was calculated at 82% (95% CI: 72–92%) based on six studies. Regarding post-exposure prophylaxis (PEP) with MVA-BN, the vaccine efficacy against Mpox was 20% (95% CI: -24–65%) from seven studies. All vaccine efficacy estimates were derived from random-effects meta-analysis.

In terms of study quality, 18 out of 33 (55%) were rated as poor, 3 out of 33 (9%) as fair, and 12 out of 33 (36%) as good. Notably, studies included in the meta-analysis generally exhibited higher quality, with 11 out of 16 (69%) rated as good.

### 5.2. Various Therapies for the Management of Mpox

#### 5.2.1. Tecovirimat

Tecovirimat (TPOXX, ST-246) is generally used to treat smallpox, as recommended by the Food and Drug Administration (FDA), but under the Expanded Access Investigational New Drug (EA-IND) protocol, it can also be used to treat Mpox. In the United States or Canada, Tecovirimat is not approved for use against Mpox, although tecovirimat is now approved in Europe for the treatment of Mpox [95,96]. Various clinical trials are being conducted to evaluate the efficacy of tecovirimat, but it is not clear.

Tecovirimat is a small molecule virus inhibitor with strong activity against Mpox and other smallpox viruses. Tecovirimat’s mechanism involves targeting the highly conserved orthopoxvirus F13L protein. This protein is essential for the creation of enveloped virions, which are necessary for viral spread. Tecovirimat functions by acting as a molecular binder, promoting the dimerization of F13L and thereby inhibiting its normal activity [97].

Early studies have indicated that tecovirimat did not significantly shorten the duration of monkeypox skin lesions [98,99]. Initial findings from the STOMP trial, which spanned multiple countries, failed to demonstrate a notable impact of tecovirimat on the healing time of skin and mucosal lesions in individuals with mild to moderate monkeypox. Notably, in the STOMP trial, a majority of participants began tecovirimat treatment more than five days after experiencing initial symptoms [100]. According to another study [101], tecovirimat, which inhibits the formation of p37 (a protein that helps in the release of enveloped virus and also enhances the virulence factor), was found to be effective in treating Mpox.

Tecovirimat is an inhibitor of the pox virus; however, Mpox also shows resistance against many drugs in cell culture, including CMX001 and ST-246 [102]. The clinical study of Mpox was done in France in 2022, where Mpox was isolated and sequenced. This study reveals that the effective concentration of tecovirimat against Mpox is in nanomolar concentrations, while cidofovir is only effective at micromolar concentrations [103]. Therefore, combination therapy may be effective for the treatment of Mpox.

The efficacy of tecovirimat has been evaluated in vitro and in vivo against a clade 2 Canadian 2022 isolate of Mpox isolated during the 2022 outbreak, and it was found that tecovirimat prevented Mpox replication in vitro with an effective concentration in the nanomolar range. The efficacy of tecovirimat was also evaluated in the CAST/EiJ mouse model with the 2022 Canadian isolate. The experiments also show that tecovirimat reduced viral titers after 1–2 weeks of infection. In 2022, tecovirimat was recommended against Mpox treatment. In vitro testing has been performed against 2022 Canadian isolates of Mpox designated Mpox/SP2833. The EC_50_ and EC_90_, for TPOXX were determined against Mpox/SP2833, a clade 1 virus in Vero E6 cells is 0.008 ± 0.0014 (EC_50_ value (μM) ± SE) and 0.0145 ± 0.0138 (EC_90_ value (μM) ± SE) [104].

In a recent study published in the Lancet journal in 2025, the author conducted an in vitro analysis comparing the susceptibility of clades 1b and 2b to tecovirimat. The clade 1b isolate was obtained in Sweden in August 2024 from a traveler returning from Africa. For comparison, the clade 2b virus was isolated in France in June 2022 from a patient at the Institut Pasteur Medical Center. Additionally, ancestral clades 1a and 2a were included as controls, utilizing U2OS cells, known for their high sensitivity to Mpox virus infection, as target cells. The findings demonstrated that tecovirimat effectively suppressed all four viral strains, exhibiting comparable half-maximal inhibitory concentrations (IC50s) ranging from 15.3 nM to 25.8 Nm [105].

#### 5.2.2. Cidofovir and Brincidofovir

Brincidofovir and cidofovir are also being used as DNA polymerase inhibitors for Mpox treatment, despite not being approved by the FDA. In vitro and in vivo studies reported that both cidofovir and brincidofovir can stop the replication of Mpox [106]. When Brin cidofovir derivative (CMX001) is given to the host, the lipid part of the drug breaks down and releases cidofovir, which forms cidofovir-diphosphate after phosphorylation, which inhibits DNA polymerase [107,108].

In a 2020 study, another new drug was reported, which is Nioch-14, having antiviral activity against Mpox [106].

#### 5.2.3. Resveratrol

A recent study indicated that resveratrol plays an important role in Mpox treatment. Resveratrol (3,5,4′-trihydroxystilbene) is a natural polyphenol found in grapes, peanuts, pines, and berries [109,110]. It is also available in red and white wines, but a higher concentration is found in red wine. The maximum amount of resveratrol is found in Polygonum cuspidatum (524 μg/g). It is also known as Japanese knotweed, which has been extensively used for inflammatory treatments in 2012–13. Resveratrol is found in cis and trans forms, but the trans form is highly effective in anti-inflammatory activity. The antiviral effects of resveratrol have been explored in several viruses, including the Mpox virus, the influenza A virus [111] Epstein–Barr virus [112], Herpes simplex virus [113], HIV [114], and hepatitis C virus [115].

Resveratrol inhibits the replication of many viruses [111,115,116,117,118,119,120]. The antiviral mechanisms of resveratrol against various types of viruses are different. Resveratrol reduced the virus yield by more than 120-fold at a concentration of 50 mM. The effect of resveratrol against Mpox has also been examined using HeLa cells. HeLa cells were infected with two strains of Mpox virus, Mpox-WA and Mpox-ROC, and it was found that resveratrol at 50 mM concentration reduced the yield of Mpox-WA and Mpox-ROC Clades by 195- and 38-fold, respectively [121].

Apart from these potential therapies, some other alternative treatments such as TOP1 inhibitor, chlorhexidine, and cysteine proteinase inhibitors are being explored, but more investigation is required for the effective treatment of these compounds.

#### 5.2.4. Plant Metabolite as a Therapeutic Drug

A recent study was conducted in 2023, in which the anti-monkeypox capability was evaluated in 56 plant products. Among these metabolites, curcumin showed the strongest binding affinity with a value of −37.43 kcal/mol, followed by gedunin (−34.89 kcal/mol), piperine (−34.58 kcal/mol), and coumadin (−34.14 kcal/mol) [122]. The study that occurred in 2022 shows various natural compounds obtained from Plantago lanceolate targeting on profilin-like protein A42R of Mpox virus [123]. The A42R protein of Mpox has an amino acid sequence similarity with profilin proteins of eukaryotic cells [124]. Profilin family proteins are actin-binding proteins responsible for F-actin assembly in the actin filament and also participate in the regulation of F-actin [125]. Silico-based screening identified various important compounds, such as luteolin 7,30-diglucuronide, luteolin 7-glucuronide-30-glucoside, plantagoside, narcissoside, and others [123]. The molecular dynamics simulation and post-simulation analysis revealed that plantagoside and narcissoside were found to be more stable products that were able to bind with the binding pocket of the viral protein of the Mpox virus due to hydrophobic and hydrogen interactions.

## 6. Conclusions

Mpox, while historically less prevalent than other orthopoxviruses, has demonstrated a significant emergence for globally, highlighting the importance of robust surveillance and preparedness. Its complications, while often self-limiting, can be severe, particularly in immunocompromised individuals and children. Transmission, primarily through close contact, necessitates stringent public health measures to curtail outbreaks. Accurate and timely diagnosis, facilitated by PCR testing, is crucial for effective management and containment. While specific antiviral treatments are available, their accessibility and widespread use remain limited, emphasizing the need for further research and development. Effective public health strategies, including vaccination and education, are essential in mitigating the impact of Mpox and preventing future pandemics. Continued research into the virus’s evolving epidemiology, transmission dynamics, and optimal treatment strategies is imperative to safeguard global health.

The 2019 coronavirus pandemic demonstrated that disease outbreaks extend beyond physical illness, significantly affecting mental well-being. Individuals experienced heightened fear, including anxiety about potential loss and depression linked to isolation. Healthcare workers facing direct exposure reported particularly elevated levels of fear. Parallels can be drawn with the Mpox outbreak, which has also been associated with notable economic and behavioral shifts, manifesting in increased anger, frustration, depression, and anxiety.

The health care provider also has very little knowledge regarding the treatment of Mpox, so proper guidance and care of Mpox-affected people should be done for the management of mental health issues in COVID-19-affected people, WHO issued advisories that may be implemented in Mpox cases.

The important suggestions to control mental disorders during the monkeypox outbreak are as follows: (a) Always study and gain knowledge from an authorized source, such as WHO. (b) Do not come under the influence of people who are already suffering from any mental disorder because of an outbreak. (c) Avoid drinking alcohol or smoking during an outbreak because it will only ruin your health condition. (d) Hand hygiene practices should be followed regularly. (e) Eat a well-balanced diet, and try to avoid sitting alone. Even when you are alone in the house, try to chat with your friends or family members via social media. (f) Exercise daily.

The outbreaks in 2022 across the globe have emphasized the significance of continuous and focused intensive care, along with the development of novel drugs.

The limited smallpox vaccine administration and decreasing immunity in the population can enhance virus spreading globally. Patients of Mpox have different kinds of complications, such as bacterial infection of the skin, skin scarring, permanent corneal scarring, pneumonia, dehydration, sepsis, and encephalitis. It is imperative to provide comprehensive care for patients, including education, nutritional support, hydration, and protection of sensitive organs such as the eyes and genitals. Therefore, it is important for the medical professional team, including clinicians, health experts, and nurses, to immediately identify infection in humans and animals, use protective measures, and initiate public health reporting to prevent an outbreak. 

## Data Availability

Not applicable.
